# Revitalizing traditional Turkish tort cheese into spreadable form: Enhancing bioactive and sensory attributes with utilization of carob molasses pulp that is a food waste

**DOI:** 10.1002/fsn3.4239

**Published:** 2024-06-11

**Authors:** Çağla Özbek, Yüksel Özdemir

**Affiliations:** ^1^ Department of Gastronomy and Culinary Arts Toros University Mersin Turkey; ^2^ Department of Nutritional and Dietetics Toros University Mersin Turkey

**Keywords:** carob molasses pulp, cream‐like cheese, enriched food, product development, traditional whey cheese, waste management

## Abstract

Tort cheese is a traditional cheese that is a gastronomically valuable product, especially in the Mediterranean Region of Turkey, obtained by prolonged boiling of whey, which is one of the most important dairy byproducts. Due to the difficulty of production and its short shelf life, this cheese, which is produced in limited quantities and has low nutritional value, is among the forgotten products. In this study, a spreadable new cheese formulation was developed to increase the edibility and nutritional value of traditional Tort cheese. In this context, pulp (carob molasses pulp (CMP)) from carob molasses production was added to the cheese in different proportions (5%, 10%, and 15%), and some physicochemical, textural, and sensory properties of the cheeses were examined. As a result of the research, it was determined that the addition of CMP caused a decrease in the pH, fat, and protein content of the cheeses, while significantly increasing the dry matter, acidity, ash, carbohydrate, antioxidant activity, and total phenolic content. With CMP addition, the hardness and water‐holding capacity of the cheeses increased, while spreadability, adhesiveness, and syneresis decreased. As the concentration of CMP increased, the L* and b* values decreased, while the a* value increased. In terms of sensory properties, the least preferred sample was the control, while the sample with 5% CMP addition, which is the most spreadable cheese, was the most preferred. As a result, a new product with lower fat content that was more durable and stable, rich in bioactive properties, and with improved sensory attributes was developed.

## Graphical abstract

The primary objective of this research is to render Tort cheese, a forgotten traditional Turkish cheese, more palatable, particularly to children. To achieve this goal, a new formulation has been developed by enriching spreadable cheeses with pulp (carob molasses pulp (CMP)), a byproduct of carob molasses production, thus providing an alternative to traditional spreadable cheese. A new product with lower fat content, more durable and stable, rich in bioactive properties, and with all sensory attributes more improved was developed and consequently, this approach not only economically valorizes a food waste but also produces a new dairy product with an enriched composition.

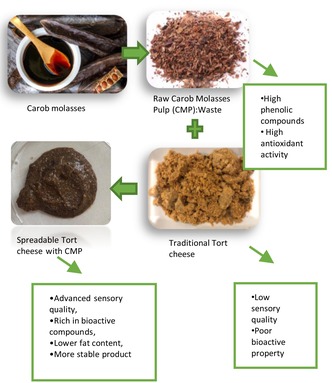

## 1 INTRODUCTION

**FIGURE 1:**
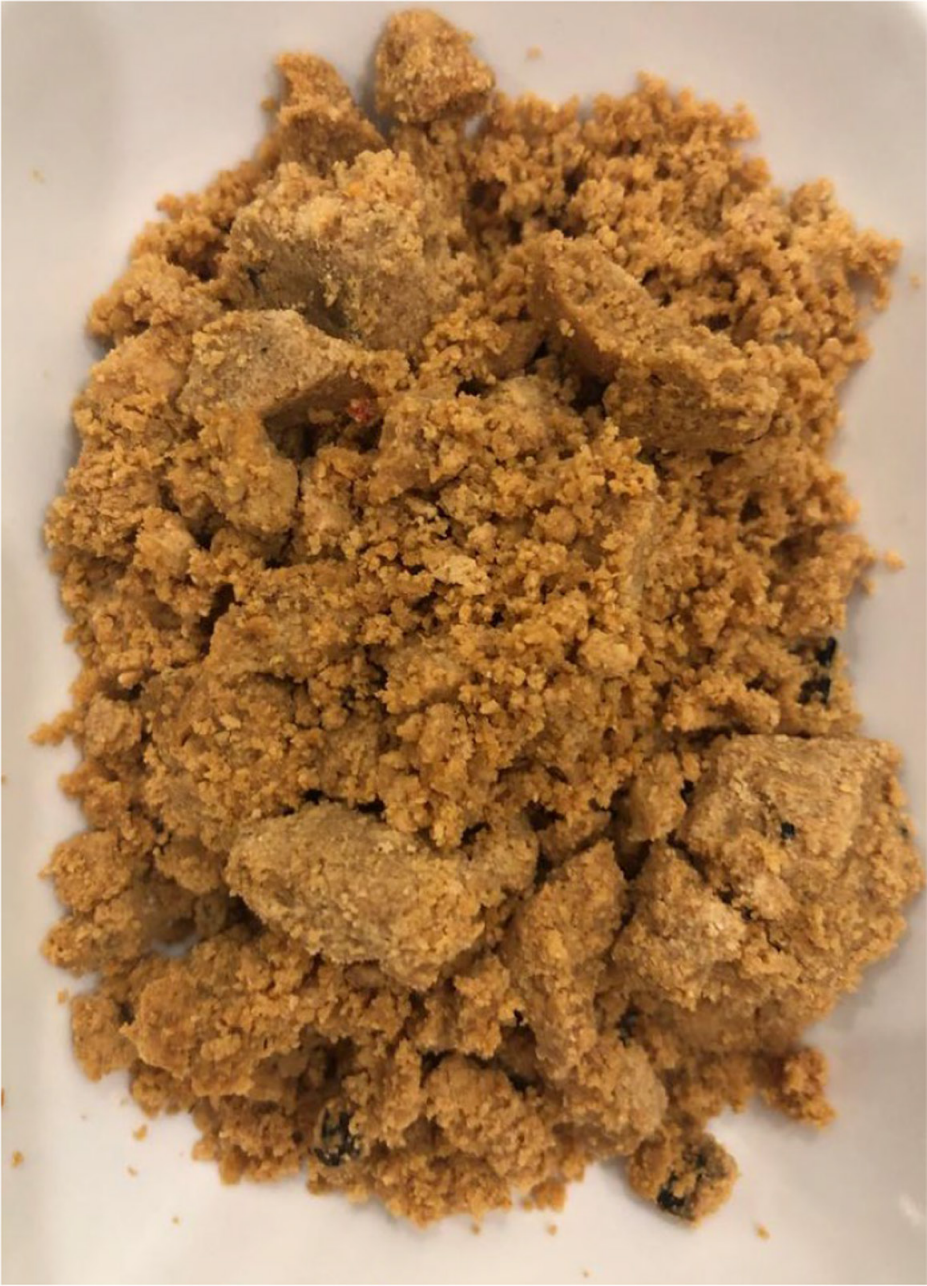
Traditional Tort cheese.

Tort cheese (Figure 1) is a dark or light brown local cheese obtained by long duration boiling (4–6 h) of the white cheese whey produced from sheep, goat, and cow milk in the Mediterranean Region of Turkey. This cheese, which is obtained by cooling at 20°C and straining from cloth bags after boiling, is produced in small‐scale family businesses. It may be stored in goat skin bags (tulum) and plastic drums at 4°C for 3–4 months, or may be consumed as fresh (Şimşek & Sağdıç, 2006). The shelf life of fresh Tort cheese is 15 days at 4°C. Tort cheese, which is in the class of semi‐fat and soft cheese, is produced by nomads living especially in the Lakes Region of Isparta, Antalya, and Afyon (Kahraman Avcı et al., 2021). While it is known as Tort or Dolaz in the Lakes region, it is also called Sarı Keş in Anamur and Horç cheese in Silifke (Kalender & Güzeler, 2013). Tort cheese is also similar to Norway's traditional cheese Mysost in terms of production method and color (Lokmanoğlu, 2020). It is generally consumed by the locals as plain for breakfast, used to make scrambled eggs, or to make a wheat dish. It is the most important food of the shepherds in the mountains, together with dry onions, embers, and village dough (Yerli et al., 2018). It contains an average of 20.25% fat, 14.10% protein, and 147.69 mg/100 g cholesterol (Dönmez et al., 2005). Components such as acetaldehyde, diacetyl, 1‐butanol, and acetone, which are known as the main aromatic components in cheeses, were determined as minor aromatic components of Tort cheese (Kahraman Avcı et al., 2021).

Tort cheese is a type of cheese that is very difficult to produce and is consumed in a limited region. In addition, cheese is less nutritious. However, Tort cheese, which is a traditional cheese that has been forgotten over time, is very important for the people of the region. In addition, the use of whey, which is one of the most important dairy wastes, in the production of this Tort cheese is important in terms of environmental and economic sustainability. The dissemination, consumption, and promotion of this cheese are important for the sustainability of gastronomy.

It was observed that the research on Tort cheese was generally carried out to determine the characteristic features of cheeses produced in certain regions (Dönmez et al., 2005; Okur & Güzel‐Seydim, 2011a, 2011b; Şimşek & Sağdıç, 2006). However, it was not found in the literature that Tort cheese was developed or used to obtain a new functional product. The consumption of cheese, which is not used in the production of a new product and whose diversity has not been increased, has remained limited. Since the milk is boiled for a long time during the production of Tort cheese, the taste and aroma of the cheese are not intense. For this reason, various additives can be used both to make cheese more delicious and aromatic and to enrich it nutritionally. For this purpose, especially the evaluation of food waste as an additive is very important in terms of environmental health and sustainability.

Carob molasses pulp (CMP) is a food waste left over from molasses (pekmez) production, which is one of the important traditional products of Turkey. It is not consumed as food alone, but rather it is widely used as animal feed (Özdemir, Özbek, et al., 2022). CMP is a product with high nutritional value and bioactive properties, which also has high antioxidant properties (Özdemir, Öncel, e al., 2022). In addition, CMP can be used as a raw material in different food products, since it does not have any negative effects on the sensory properties of foods (Özdemir et al., 2021).

In this study, spreadable Tort cheese with CMP flour was produced. Thus, the aim of this study was to increase its consumption by developing a new product that children would love to consume due to its spreadable chocolate‐like texture and color. In addition, increasing the nutritional value of cheese and strengthening it in terms of taste and aroma were among the main objectives. Additionally, CMP flour, which is a food waste, was evaluated for the purpose of developing a new product, and it was aimed to gain environmental and economic sustainability.

## 2 MATERIALS AND METHODS

### 2.1 Raw materials

Tort cheese was procured from a local producer in Anamur province in Mersin, Turkey. Carob molasses pulp (CMP) was supplied by Atışeri company (Mersin, Turkey), which produces carob molasses. Pasteurized milk (3% fat, 2.8% protein), milk cream (70% fat), sodium caseinate, and granulated sugar (refined beet sugar) used in the production of spreadable cheese were procured daily from the local markets and the products were stored at +4°C for further use.

### 2.2 CMP flour production

Carob molasses production (CMP) was dried in an oven (JP Selecta, Spain) at 50°C, until the moisture content was less than 10%. Then, it was ground in a laboratory mill (Model 4‐E; Miksan, Istanbul, Turkey) and passed through sieves (Model VE 100; Retsch, Germany) with a pore diameter of 100 μm. The obtained flour was used to make spreadable cheese.

### 2.3 Spreadable Tort cheese production

**FIGURE 2:**
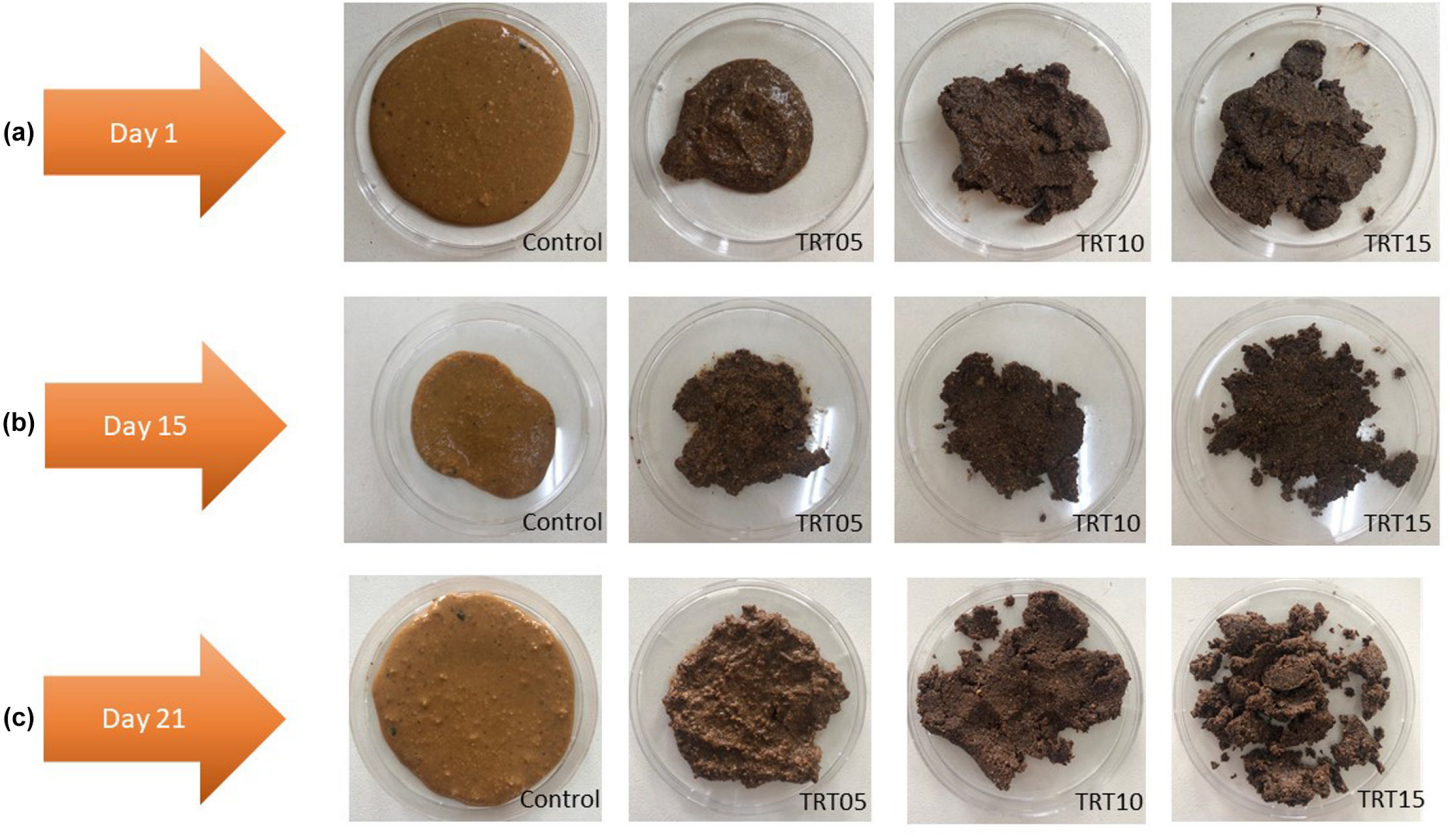
(a) Spreadable Tort cheese samples on the 1st day of storage. (b) Spreadable Tort cheese samples on the 15th day of storage. (c) Spreadable Tort cheese samples on the 21st day of the storage, CMP concentrations of cheese samples are 0% for control, 5% for TRT05, 10% for TRT10, and 15% for TRT15.

For the production of spreadable Tort cheese, all raw materials were mixed with a hand‐type mixer (Braun MultiQuick, 7 MQ7035I, Germany) in specified amounts mentioned in Table 1. Then the mixture was blended with an Ultra Turrax blender (Janke & Kunkel KG, IKA Werke, Germany) until it became homogeneous and heated to 70°C for 2–3 min. After heat treatment, the cheese was cooled to room temperature and filled into the glass jars with 175‐g capacity. The cheeses were stored at 4 ± 1°C for 21 days. Cheese analyses were performed during the 1st, 15th, and 21st days of storage. The visuals for each storage day of the samples are provided in Figure 2.

**TABLE 1:** Production formulations of spreadable Tort cheese.

Sample number	CMP%	Ingredients (g)					
		Tort cheese	Pasteurized milk	Milk cream	Sodium caseinate	Granulated sugar	CMP
Control	0	56	25	19	1	3	0
TRT05	5	56	25	19	1	3	5.5
TRT10	10	56	25	19	1	3	11.5
TRT15	15	56	25	19	1	3	18.4

Abbreviation: CMP, Carob molasses pulp.

### 2.4 Spreadable Tort cheese analysis

#### 2.4.1 Physicochemical analysis

The pH of the cheese was determined using a pH meter (inoLab pH 720, WTW GmbH, Weilheim, Germany) (Hayaloğlu et al., 2011). The alkali titration method was used to determine acidity, and the results were expressed as a percentage of lactic acid (Metin, 2009). The dry matter content was detected by the gravimetric method (IDF, 2004), protein content was analyzed by the Kjeldahl method (IDF, 2014), and the fat content was detected by the Soxhlet extraction method (Buhler et al., 2020). The ash content was determined by a gravimetric method (Kurt et al., 2007). Total carbohydrate content was determined according to the phenol–sulfuric acid method (Karadeniz et al., 2021).

#### 2.4.2 Total phenolic compounds and antioxidant activity

Total phenolic compounds were determined by the Folin–Ciocalteu method (Mediza Romero et al., 2021). According to this method, the mixture of 1‐gram cheese sample and 25 mL of 80% methanol solution was centrifuged (Nuve NF800 R, Turkey) at 4500 rpm for 15 min. Then, 0.2 mL of the supernatant was taken and mixed with 1.5 mL of Folin–Ciocalteu reagent (reagent:water mixture, 1:10 v/v). After keeping this solution in the dark for 5 min, 1.5 mL of 7.5% sodium carbonate solution was added. After keeping the solution in the dark place for 90 min, the absorbance was measured at 765 nm in a ultraviolet–visible (UV–Vis) spectrophotometer (UV‐1601; Rayleigh, BFRL, China), and the results were expressed as milligrams of gallic acid per liter (mg gallic acid/L).

Antioxidant activity was analyzed, according to Trentin et al. (2022). For this purpose, 5 g of cheese was mixed with 80% methanol solution for 30 min in a magnetic stirrer (Digitmex, Hong Kong). The mixture was centrifuged at 4500 rpm for 15 min after filtration. Then, 100 μL of the extract was taken into a cuvette and 3900 μL of DPPH (1,1‐diphenyl‐2‐picrylhydrazyl radical) solution (3.94 mg/100 mL methanol) was added. This solution was kept in the dark for 30 min and the absorbance was measured at 515 nm on a UV–Vis spectrophotometer (UV‐1601; Rayleigh, BFRL, China). Trolox calibration was used to determine the antioxidant activity (free radical‐scavenging activity) and the results were expressed as gram Trolox equivalents per liter (gTE/L).

#### 2.4.3 Texture analysis

A TA.XTPlus Texture Analyzer (Stable Micro Systems Ltd., Godalming, Surrey, UK) was used to examine the textural properties. For the texture profile analysis (TPA), cheese samples were taken in a cylinder that had 70‐mm diameter and 60‐mm height and with completely smooth and uniform surfaces. A cone probe fitted with a 45° (TA15/100, 30 mm in diameter) and 4.5 kg load cell was used for the measurement. Cheese samples were evaluated in terms of hardness, spreadability, and adhesiveness. Data collection and calculation were conducted using Exponent Stable Micro Systems Version 6.1.16.0 equipment software (Stable Micro Systems Ltd., Godalming, Surrey, UK) (Ghorbel et al., 2016).

#### 2.4.4 Serum separation (syneresis) and water‐holding capacity

Syneresis was determined by weighing the amount of serum (g) filtered through filter paper in 120 min for 25 g sample at 4 ± 1°C, and the results were expressed as percentages (Hassan et al., 2024). A 5 g sample was weighed for moisture‐holding capacity determination and then centrifuged at 4500 rpm and 10°C for 30 min. Subsequently, the supernatant was removed, and the pellet was weighed to calculate the water‐holding capacity (Hamdy et al., 2021).

#### 2.4.5 Color measurement

Color analysis was performed using a colorimeter (HunterLab., Hunter Associates Laboratory, Reston, VA, USA). The colorimeter was calibrated with reference to white before measurements. Results were explained according to parameters L*, a*, b*, °hue, and chroma (Milovanovic et al., 2020).

#### 2.4.6 Sensory evaluation

Sensory analyses were carried out by a group of 13 trained panelists (mean age 25, nonsmokers) consisting of lecturers and students from Toros University, Gastronomy and Culinary Arts Department. The samples were served at room conditions with a slice of bread and water. The cheese samples were evaluated in terms of external appearance, color, odor, creaminess, firmness, spreadability, taste, and overall acceptability by applying the 9‐point (1: extremely dislike to 9: extremely like) linear hedonic scale scoring test (Metin, 2009).

### 2.5 Statistical analysis

SPSS (Version 20, IBM, USA) statistics software's one‐way and two‐way analyses of variance (ANOVAs) were employed for the statistical analysis of the data. Duncan's multiple comparison tests were used to evaluate differences among the cheese samples (*p* < .05) and to determine the Duncan correlation coefficients with 95% confidence level (Guo et al., 2018).

The Minitab 18 software was employed to conduct the principal component analysis (PCA) on a matrix comprising eight sensory attributes, namely appearance, color, odor, creaminess, firmness, spreadability, taste, and overall acceptability. This approach aimed to assess the primary patterns of variation in cheeses and their storage conditions. Subsequently, a factorial analysis was conducted on the two most significant principal components.

## 3 RESULTS AND DISCUSSION

### 3.1 Physicochemical properties of spreadable Tort cheese

The physicochemical properties of spreadable Tort cheese are presented in Table 2. While the pH, titratable acidity, and dry matter content of the samples were investigated during a 21‐day storage period, the protein, fat, ash, and total carbohydrate contents were determined on the 1st day of storage. It was observed that the addition of CMP significantly reduced the pH of the cheeses throughout the storage period and increased their acidity on the 1st day of storage (*p* < .05). In the study conducted by Öncel and Özdemir (2023), it was stated that the metabolic activities of lactic acid bacteria (LAB) in yogurt increased with the addition of CMP and thus they detected an increase in acidity level and a decrease in pH values. Similar results were observed when carob flour or juice was added to dairy products (Akdeniz, 2023; Atasoy, 2009). As the storage progressed, a decrease in pH values in all cheese samples and an increase in acidity in control were observed (*p* < .05), which may be related to proteolysis, lipolysis, or protein denaturation that occurred during storage (Aydemir, 2018). The fact that a significant increase in acidity was detected only in the control sample indicates that the addition of CMP stabilizes the acidity development in cheeses. While the addition of CMP significantly increased the dry matter content of the cheeses as expected (*p* < .05), no significant changes were observed in the dry matter content of the cheeses during storage (*p* > .05). The protein content of spreadable Tort cheeses ranged from 9.18% to 9.85%, while the fat content spreadable Tort cheeses ranged from 13.08% to 15.08%. It is reported that traditional Tort cheese contains 20.25% fat and 14.10% protein, respectively (Dönmez et al., 2005). Due to the pasteurized milk and cream used to make Tort cheese spreadable, the total fat and protein ratios of spreadable Tort cheeses were found to be lower compared to traditional cheeses. As CMP addition increased, the protein and fat ratios of the cheeses decreased significantly (*p* < .05). This decrease occurred due to an increase in total solids content with CMP addition. The addition of CMP significantly increased the ash and total carbohydrate content of the cheeses (*p* < .05). It is known that the ash amount of CMP is 2.56% and the total carbohydrate amount in the dry matter content is 5.92% (Özdemir, Özbek, et al., 2022).

**TABLE 2:** Physicochemical properties of spreadable Tort cheese (*n* = 3).

Physicochemical properties	Storage day	Sample number			
		Control	TRT05	TRT10	TRT15
		0% CMP	5% CMP	10% CMP	15% CMP
pH	Day 1	5.93 ± 0.04^aK^	5.39 ± 0.05^bK^	5.07 ± 0.07^cK^	4.97 ± 0.01^dK^
	Day 15	5.51 ± 0.03^aL^	5.02 ± 0.05^bL^	4.94 ± 0.01^dL^	4.81 ± 0.01^cK^
	Day 21	4.94 ± 0.00^aM^	4.83 ± 0.07^bM^	4.76 ± 0.05^bL^	4.76 ± 0.04^bL^
Total acidity (Lactic acid %)	Day 1	0.84 ± 0.02^aM^	1.18 ± 0.01^cK^	1.17 ± 0.01^cK^	0.21 ± 0.01^bK^
	Day 15	1.17 ± 0.14^aL^	1.20 ± 0.11^aK^	1.23 ± 0.06^aK^	1.33 ± 0.13^aK^
	Day 21	1.46 ± 0.09^aK^	1.28 ± 0.27^aK^	1.31 ± 0.19^aK^	1.46 ± 0.13^aK^
Dry matter (%)	Day 1	47.20 ± 0.07^dK^	49.60 ± 0.14^cL^	56.48 ± 0.30^bK^	59.29 ± 0.12^aK^
	Day 15	46.33 ± 0.32^dK^	50.52 ± 0.07^cK^	56.50 ± 0.33^bK^	57.58 ± 0.09^aK^
	Day 21	47.50 ± 0.29^dK^	51.28 ± 0.16^cK^	54.50 ± 0.21^bK^	58.57 ± 0.10^aK^
Protein (%)	Day 1	9.85 ± 0.11^a^	9.61 ± 0.06^b^	9.34 ± 0.08^c^	9.18 ± 0.10^c^
Fat (%)	Day 1	15.08 ± 0.18^a^	14.48 ± 0.20^b^	13.60 ± 0.20^c^	13.08 ± 0.06^d^
Ash (%)	Day 1	2.83 ± 0.06^c^	2.95 ± 0.01^b^	3.04 ± 0.01^a^	3.05 ± 0.01^a^
Total carbohydrate (g glucose/100 mL)	Day 1	0.74 ± 0.08^b^	0.87 ± 0.08^ab^	0.93 ± 0.11^a^	0.98 ± 0.09^a^

*Note*: ^a–d^Values that are shown in the same line with different superscript letters are comparing the effect of CMP concentration at each storage day and are different at the *p* < .05 level of significance. ^K–M^Values that are shown in the same column with different superscript letters are comparing the effect of storage time at each CMP concentration and are different at the *p* < .05 level of significance.

Abbreviation: CMP, Carob molasses pulp.

### 3.2 Total phenolic compounds and antioxidant activity

The total phenolic content and antioxidant activities of spreadable Tort cheeses are presented in Table 3. The addition of CMP notably increased the phenolic content and antioxidant activity of the cheeses (*p* < .05). As the concentration of CMP increased, significant enhancements in these properties were observed (*p* < .05). CMP contains 3.05% phenolic content and 0.91% antioxidant activity on a dry‐weight basis (Özdemir, Özbek, et al., 2022). Carob pods contain various phenolic compounds, such as gallic acid, flavonoids, syringic acid, quercetin, rutin, myricetin, catechin, and epicatechin (Goulas & Georgiou, 2020). The incorporation of CMP enriched the total phenolic content and antioxidant activity of spreadable Tort cheeses due to its phenolic compound ingredient.

**TABLE 3:** Total phenolic compounds and antioxidant activity of spreadable Tort cheese (*n* = 3).

Bioactive properties	Storage day	Sample number			
		Control	TRT05	TRT10	TRT15
		0% CMP	5% CMP	10% CMP	15% CMP
Total phenolic compounds (mg GAE/L)	Day 1	494.00 ± 6.67^cK^	619.00 ± 1.67^bK^	669.00 ± 5.00^bK^	762.33 ± 1.67^aK^
	Day 15	227.33 ± 3.33^dL^	290.78 ± 0.19^cL^	364.00 ± 3.33^bL^	685.67 ± 5.00^aL^
	Day 21	175.67 ± 1.67^dL^	245.67 ± 1.67^cM^	340.78 ± 0.19^bM^	594.33 ± 0.58^aM^
Antioxidant activity (g/100 g)	Day 1	5.01 ± 0.00^dM^	11.65 ± 0.00^cM^	12.53 ± 0.00^bM^	12.69 ± 0.00^aL^
	Day 15	7.58 ± 0.00^dL^	12.47 ± 0.02^cL^	12.79 ± 0.00^bK^	12.81 ± 0.00^aK^
	Day 21	9.14 ± 0.00^dK^	12.58 ± 0.01^cK^	12.73 ± 0.00^bL^	12.81 ± 0.01^aK^

*Note*: ^a–d^Values that are shown in the same line with different superscript letters are comparing the effect of CMP concentration at each storage day and are different at the *p* < .05 level of significance. ^K–M^Values that are shown in the same column with different superscript letters are comparing the effect of storage time at each CMP concentration and are different at the *p* < .05 level of significance.

Abbreviation: CMP, Carob molasses pulp.

During storage, a decrease in the total phenolic content of spreadable Tort cheeses was noted (*p* < .05). Factors, such as oxidation, enzymatic activity, pH changes, interactions with other components, microbial activity, and storage conditions, might contribute to the decline in phenolic content during storage (Amarowicz et al., 2009). In the current study, the observed significant decrease in pH values throughout the storage period was thought to be closely associated with the reduction in the phenolic compound content across all samples. Conversely, the antioxidant activity of spreadable Tort cheeses increased throughout the storage period (*p* < .05). This development could be attributed to the formation of certain antioxidant compounds, such as melanoidins, amadori compounds, hydroxymethylfurfural, pyrazines, and pyrrolesresulting from the Maillard reaction (Shakoor et al., 2022), occurring during Tort cheese production (Kahraman Avcı et al., 2021) and persisting during storage (Spanneberg et al., 2012). Furthermore, the breakdown of proteins to peptides in the cheeses during storage might also lead to the formation of certain antioxidant compounds (Santiago‐López et al., 2018).

### 3.3 Texture analysis

The textural properties of spreadable Tort cheeses are presented in Table 4. It was evident that the addition of CMP increased hardness while decreasing spreadability and adhesiveness (*p* < .05). Hardness refers to the resistance of a material to deformation or penetration. Spreadability can be defined as the ease with which a substance can be spread or smeared over a surface. Adhesiveness is defined as the tendency of a substance to stick to another surface upon contact (Çağlar et al., 2023). The increase in dry matter, particularly due to the increase in carbohydrates with the addition of CMP, resulted in increased hardness (*p* < .05). As hardness increased, a decrease in adhesiveness and spreadability also occurred accordingly (*p* < .05). Similarly, CMP addition increased the hardness in cake samples (Özdemir et al., 2023) while reducing spreadability in wafer cream (Özdemir, Öncel, et al., 2022). Çağlar et al. (2023) also reported that as hardness increased, spreadability and adhesiveness decreased.

**TABLE 4:** Textural properties of spreadable Tort cheese (*n* = 3).

Sample number	CMP%	Textural properties		
		Hardness (g)	Spreadability (g.s)	Adhesiveness (g.s)
Control	0	58.67 ± 3.51^c^	13,554 ± 1193^a^	332.67 ± 11.37^a^
TRT05	5	76.33 ± 7.50^b^	11,390 ± 1006^b^	265.00 ± 18.33^b^
TRT10	10	147.00 ± 9.54^a^	8442 ± 776^c^	187.33 ± 10.60^c^
TRT15	15	153.00 ± 4.58^a^	5804 ± 211^d^	144.67 ± 36.09^d^

*Note*: ^a–d^Values that are shown in the same column with different superscript letters are comparing the effect of CMP concentration at the first storage day and are different at the *p* < .05 level of significance.

Abbreviation: CMP, Carob molasses pulp.

### 3.4 Serum separation (syneresis) and water‐holding capacity

**FIGURE 3:**
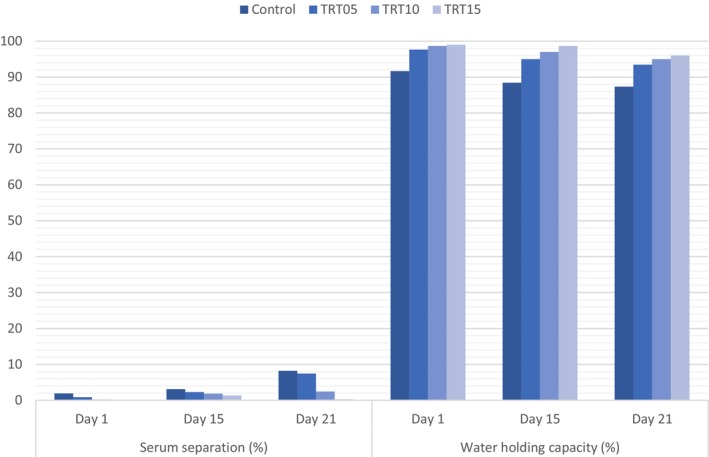
Serum separation and water‐holding capacities of spreadable Tort cheese (*n* = 3), CMP concentrations of cheese samples are 0% for control, 5% for TRT05, 10% for TRT10, and 15% for TRT15.

Serum separation during storage is a key textural attribute affecting consumer acceptance of spreadable dairy products. The data representing serum separation and water‐holding capacities of cheeses are provided in Figure 3. The use of 5% CMP in spreadable Tort cheese reduced serum separation by 54% on the 1st day of storage (*p* < .05). Serum separation amounts were reduced by 89% with 10% CMP usage and 99% with 15% CMP usage at this stage. Increasing CMP concentration significantly decreased serum separation throughout storage (*p* < .05), while significantly increasing water‐holding capacities (*p* < .05). Water‐holding capacity, defined as the tendency of fibrous hydrophilic materials to associate with water, was measured by the water held by the insoluble portions of fibrous materials (Fennema, 1996). CMP's substantial crude fiber content (29.30%) (Özdemir, Özbek, et al., 2022) indicates its water‐holding capacity. While serum separation increased significantly during storage for each cheese sample (*p* < .05), water‐holding capacities decreased significantly (*p* < .05). The expected increase in syneresis and decrease in water‐holding capacity during storage can be explained by Darcy's law, which states that the flow velocity of syneresing liquid (serum in cream cheese) is directly related to the pore size of the gel and inversely related to the viscosity of the serum phase (Fox et al., 2000).

### 3.5 Color

The color characteristics of spreadable cheeses are provided in Table 5. The L*, a*, and b* values of each sample were found statistically different from each other during the storage period (*p* < .05). It was observed that L* (whiteness) and b* (yellowness) values decreased proportionally with the increase in concentration due to the addition of CMP (*p* < .05). Carob pods contain a variety of polyphenols, tannins, and sugars, contributing to the dark brown color of carob molasses (Tounsi et al., 2020). Traditional Tort cheese has a yellowish‐brown color, and the addition of CMP darkened the color of the cheese due to the natural brown color of CMP. Similar results were observed in studies conducted by Özdemir, Özbek, et al. (2022) and Özdemir et al. (2023). The a* (redness) values increased with the addition of CMP (*p* < .05). According to IEC (1999), the color of carob molasses is wood‐black red (L* = 29.8, a* = 11.42, b* = 1.99). The natural redness carried by CMP resulted in an increase in a* in the cheeses. Similar results were also reported by Karaca et al. (2012). The storage period did not affect the color characteristics of spreadable cheeses (*p* > .05). However, the dual effect of CMP addition and storage time was found significant on L* value (*p* < .05). The hue angles of the samples changed between 52.15 (TRT10‐day 1) and 72.97 (control‐day 15) and they represents a color in the yellow/green region. Chroma gives information about the saturation of the color (Delikanli & Ozcan, 2016). The addition of CMP produced more matte color and the control was significantly more vibrant. A significant difference was observed in chroma values depending on the use of CMP in the first 15 days of storage (*p* < .05), but on the 21st day, there was no difference between the samples (*p* > .05). As the storage time increased, the opacity of the samples decreased (*p* < .05). CMP contains insoluble fiber and other particulate matter that can remain suspended in the media, leading to an opaque appearance. Additionally, CMP usage increased the viscosity of the Tort cheese, making it appear thicker and less translucent.

**TABLE 5:** Color properties of spreadable Tort cheese (*n* = 3).

Color properties	Storage day	Sample number			
		Control	TRT05	TRT10	TRT15
		0% CMP	5% CMP	10% CMP	15% CMP
L*	Day 1	23.40 ± 2.94^aK^	14.37 ± 4.87^bK^	11.78 ± 2.46^cK^	10.25 ± 3.45^cK^
	Day 15	23.30 ± 1.49^aK^	15.26 ± 1.49^bK^	12.20 ± 1.32^cK^	9.99 ± 1.40^dK^
	Day 21	23.56 ± 2.61^aK^	15.95 ± 4.82^bK^	11.62 ± 2.56^bK^	10.83 ± 6.28^bK^
a*	Day 1	5.60 ± 0.84^cK^	6.41 ± 0.36^bK^	7.64 ± 0.82^aK^	7.70 ± 0.33^aK^
	Day 15	5.86 ± 0.39^cK^	6.23 ± 0.49^bK^	7.76 ± 0.45^aK^	7.22 ± 0.68^aK^
	Day 21	5.38 ± 0.30^cK^	6.12 ± 0.72^bK^	7.13 ± 0.59^aK^	7.32 ± 1.39^aK^
b*	Day 1	24.92 ± 1.34^aK^	20.77 ± 0.69^bK^	14.51 ± 2.28^cK^	13.12 ± 0.68^cK^
	Day 15	25.77 ± 2.90^aK^	20.23 ± 0.78^bK^	13.90 ± 0.28^cK^	13.56 ± 2.36^cK^
	Day 21	24.85 ± 1.69^aK^	20.15 ± 1.55^bK^	14.24 ± 0.85^cK^	13.38 ± 6.09^cK^
Color (°hue)	Day 1	68.72 ± 0.66^aL^	64.73 ± 2.56^abL^	52.15 ± 13.08^bK^	65.76 ± 1.37^aKL^
	Day 15	72.97 ± 1.11^aK^	70.33 ± 0.74^abK^	62.35 ± 2.03^bcK^	53.06 ± 9.63^cL^
	Day 21	70.28 ± 0.84^aL^	69.27 ± 0.55^aK^	66.65 ± 2.81^aK^	66.66 ± 5.88^aK^
Chroma	Day 1	14.93 ± 1.38^aL^	10.82 ± 0.95^bM^	9.53 ± 1.22^bL^	9.99 ± 0.72^bK^
	Day 15	26.95 ± 2.88^aK^	21.48 ± 0.88^bK^	14.56 ± 0.11^cK^	10.69 ± 1.62^dK^
	Day 21	18.96 ± 1.69^aL^	17.27 ± 1.70^aL^	15.51 ± 0.71^aK^	14.43 ± 6.13^aK^

*Note*: ^a–d^Values that are shown in the same line with different superscript letters are comparing the effect of CMP concentration at each storage day and are different at the *p* < .05 level of significance. ^K–M^Values that are shown in the same column with different superscript letters are comparing the effect of storage time at each CMP concentration and are different at the *p* < .05 level of significance.

Abbreviation: CMP, Carob molasses pulp.

### 3.6 Sensory properties

**FIGURE 4:**
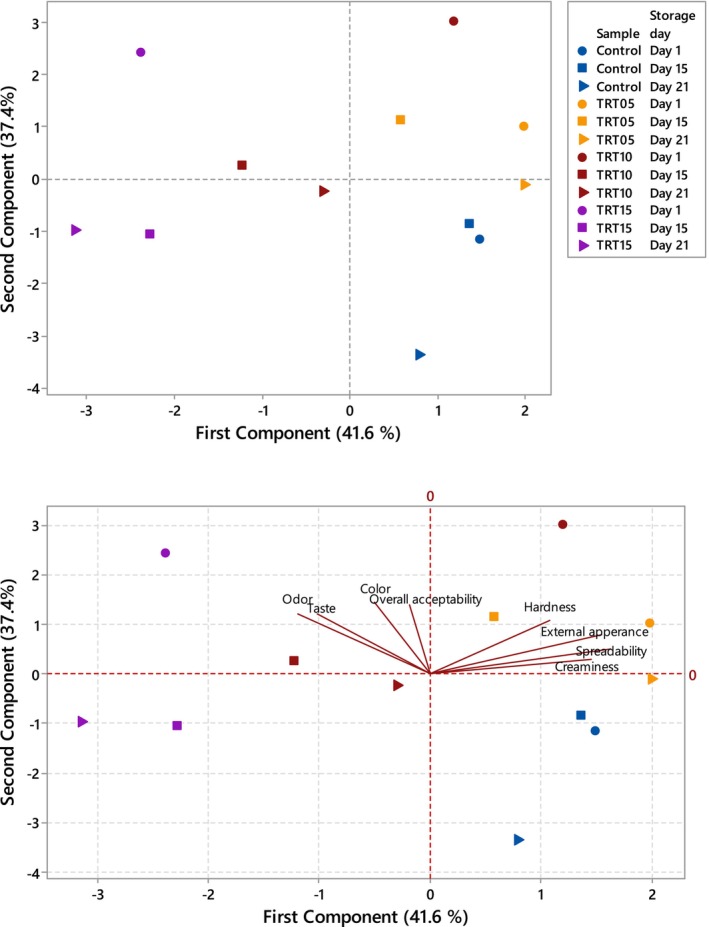
Principal component analysis biplot on sensory properties of spreadable Tort cheese (*n* = 3), CMP concentrations of cheese samples are 0% for control, 5% for TRT05, 10% for TRT10, and 15% for TRT15.

Data on external appearance, color, odor, creaminess, firmness, spreadability, taste, and overall acceptability of spreadable Tort cheeses were subjected to PCA (Figure 4). The multivariate treatment of the data obtained for the samples permitted the reduction of the variables to two principal components, which together explained 79% of the total variability. The first axis accounted for 41.6% and the second axis for 37.4%. According to the PCA biplot, hardness, external appearance, spreadability, and creaminess were positively correlated to the first principal component (PC1) axis, whereas odor, taste, color, and overall acceptability were negatively correlated to the PC1 axis. All sensory properties were positively correlated to the second principal component (PC2) axis. When examining the PCA plot, it was observed that all textural features formed one group, while odor and taste formed another group, and color and overall acceptability were closely related to each other. Spreadable Tort cheese samples were observed to differentiate from each other in terms of all sensory characteristics (*p* < .05). The best textural properties during storage were observed in cheeses with 5% CMP addition (*p* < .05). On the 15th and 21st days of storage, cheeses with 10% CMP addition were the most preferred samples in terms of taste and odor (*p* < .05). Throughout the storage period, the least preferred sample in terms of all features was the control, while cheeses with 15% CMP addition were not accepted by the panelists. Although the cheese with 15% CMP addition received the highest score in overall acceptability on the 1st day of storage (*p* < .05), as the storage progressed, the most preferred and accepted cheese in terms of all features was the sample with 5% CMP addition (*p* < .05). According to the comparison based on the two‐way ANOVA method, the dual effect of CMP addition and storage time was found significant on external appearance, color, odor, firmness, and taste (*p* < .05). As the CMP concentration used increased, the color obtained was more appreciated by the panelists (*p* < .05). Instrumental color analysis results showed that as CMP concentration increased, L*, b* hue angle, and chroma values decreased, and a* value increased. In this case, it was understood that the panelists gave higher scores to darker, reddish, and less yellow spreadable cheeses. In studies where CMP addition was made to wafer cream (Özdemir, Öncel, et al., 2022), yogurt (Öncel & Özdemir, 2023), and ice cream cone (Özdemir, Özbek, et al., 2022), it was generally reported that CMP addition did not have a negative effect on organoleptic properties of foods. In the study where CMP addition was made in the range of 5%–15% to wafer cream, it was reported that 5% CMP addition reduced the appearance scores, while increasing the concentration of CMP use significantly increased the sweetness level (Özdemir, Öncel, et al., 2022). In the other study, the difference observed in the color and appearance features of CMP‐added yogurts was liked by the panelists, and it was reported that CMP addition improved all sensory characteristics of yogurts (Öncel & Özdemir, 2023).

## 4 CONCLUSION

The primary objective of this research is to render Tort cheese, a forgotten traditional Turkish cheese, more palatable, particularly to children. To achieve this goal, a new formulation has been developed by enriching spreadable cheeses with pulp (CMP), a byproduct of carob molasses production, thus providing an alternative to traditional spreadable cheese. This approach not only economically valorizes food waste, but also produces a new dairy product with an enriched composition. When CMP was added to spreadable Tort cheese, a decrease in pH, fat, and protein ratios was observed, while an increase in dry matter, acidity, ash, and carbohydrate levels was noted. The addition of CMP contributed to the nutritional properties of the product by reducing fat content and increasing ash content, thereby enhancing mineral content. The most significant effect of CMP addition was observed on the bioactive properties of the cheeses, with a substantial increase in antioxidant activity and phenolic compound levels. While CMP addition increased the hardness of the cheeses, it decreased spreadability and adhesiveness. Syneresis was reduced by 54%–99% with CMP addition. It was found that CMP usage reduced the L* and b* values and increased the a* value. The control sample was the least preferred in terms of sensory characteristics, whereas CMP addition enhanced all sensory attributes. The most preferred sample was the one with 5% CMP addition in terms of sensory properties, especially spreadability. For further enhancement of the product functionality, future studies may explore the use of different sugar substitutes and protein enrichment.

## AUTHOR CONTRIBUTIONS

**Çağla Özbek:** Formal analysis (equal); investigation (equal); methodology (equal); software (equal); writing – original draft (equal). **Yüksel Özdemir:** Conceptualization (equal); data curation (equal); supervision (equal); validation (equal); visualization (equal).

## CONFLICT OF INTEREST STATEMENT

There are no conflicts of interest. This is an original, theoretical work that has only been submitted to this Journal.

## ETHICS STATEMENT

This study does not involve any human or animal testing.

## INFORMED CONSENT

Written informed consent was obtained from all study participants.

## Data Availability

No data were used for the research described in the article.
